# Effects of Internal Exposure of Radioactive ^56^MnO_2_ Particles on the Lung in C57BL Mice

**DOI:** 10.3390/cimb45040209

**Published:** 2023-04-06

**Authors:** Zhaslan Abishev, Bakhyt Ruslanova, Saulesh Apbassova, Nailya Chaizhunussova, Dariya Shabdarbayeva, Almas Azimkhanov, Kassym Zhumadilov, Valeriy Stepanenko, Sergey Ivanov, Peter Shegay, Masaharu Hoshi, Nariaki Fujimoto

**Affiliations:** 1 Department of Pathological Anatomy and Forensic Medicine, Semey Medical University, Semey 071400, Kazakhstan; zhaslan_love@mail.ru (Z.A.); baharuslanova@gmail.com (B.R.); saulesh.apbasova@smu.edu.kz (S.A.); dariya_kz67@mail.ru (D.S.); 2 Department of Public Health, Semey Medical University, Semey 071400, Kazakhstan; n.nailya@mail.ru; 3 National Nuclear Center of the Republic of Kazakhstan, Kurchatov 071100, Kazakhstan; azimhanov@nnc.kz; 4 Department of Nuclear Physics, L.N. Gumilyov Eurasian National University, Astana 010000, Kazakhstan; zhumadilovk@gmail.com; 5 A. Tsyb Medical Radiological Research Center—National Medical Research Center of Radiology, Ministry of Health of Russian Federation, 249031 Obninsk, Russia; valerifs@yahoo.com (V.S.); oncourolog@gmail.com (S.I.); 6 National Medical Research Center of Radiology, Ministry of Health of the Russian Federation, 249031 Obninsk, Russia; dr.shegai@mail.ru; 7 The Center for Peace, Hiroshima University, Hiroshima 730-0053, Japan; mhoshi@hiroshima-u.ac.jp; 8 Research Institute for Radiation Biology and Medicine, Hiroshima University, Hiroshima 734-0037, Japan

**Keywords:** residual radiation, internal radiation exposure, radiation injury, lung

## Abstract

The investigation of the radiation effects of the atomic bombing in Hiroshima and Nagasaki has revealed concerns about the impact of the residual radioactive dust produced in the soil. Manganese-56 is one of the major radioisotopes produced by neutrons from the bomb; hence, we previously examined the biological effects of manganese dioxide-56 (^56^MnO_2_) in Wistar rats, in which significant changes were found in the lung. In the present study, ten-week-old male C57BL mice were exposed to three doses of radioactive ^56^MnO_2_, stable MnO_2_ particles, or external γ-rays (2 Gy) to further examine the effects of ^56^MnO_2_ in a different species. The estimated absorbed radiation doses from ^56^MnO_2_ were 26, 96, and 250 mGy in the lung. The animals were examined at 3, 14, and 70 days post exposure. Histologically, no exposure-related changes were found in the lungs of any group. However, pulmonary mRNA expression of aquaporin 1, which is a useful marker for lung pathophysiology, was significantly elevated at 14 and 70 days, although no such changes were found in the mice exposed to external γ-rays (2 Gy). These data indicated that the inhalation exposure to ^56^MnO_2_ particles, with <250 mGy of organ doses, produced significant biological responses in the lung.

## 1. Introduction

The evaluation of the health effects of the atomic bomb in Hiroshima and Nagasaki in 1945 revealed concerns about the potential significance of the residual radioactivity. This is because the people who moved to these cities soon after detonation and were exposed to only residual radiation suffered from acute radiation syndromes [1]. A recent cohort study concluded that solid cancer mortality risks were significantly high among the early entrants after the Hiroshima atomic bombing, which also suggested the significance of residual radiation [2]. One of the primary radioisotopes produced in the soil by the neutron beam from the atomic bomb explosion was manganese-56 (^56^Mn) [3]. We investigated the biological effects of neutron-activated manganese dioxide-56 (^56^MnO_2_) particles in Wistar rats to understand the potential health effects caused by the residual radiation [4]. The rat dosimetry data revealed that the highest radiation doses were received in the gastrointestinal tract, followed by the skin and the lungs [5]. Our previous studies revealed that internal ^56^MnO_2_ particle exposure significantly changed gene expression with physiological importance in the lung and the male reproductive system, which indicated the potential significance of internal exposure by residual activity [6,7].

Male C57BL mice were exposed to ^56^MnO_2_ particles activated by neutrons for the first time for further investigation. The effects on the lung, which is a primary target of inhalation, were examined. Generally, high doses (>8 Gy) of thoracic radiation induce radiation pneumonitis and lung fibrosis in laboratory animals as well as in humans [8,9]. Studies have suggested the involvement of transforming growth factor-β (TGF-β) and the Smad genes (Smad2 and Smad7) in the development of those radiation-induced lung injuries [10,11]. Changes in mRNA expression of these genes could be valuable markers for lung injuries [12,13]. Additionally, the aquaporin gene (Aqp) expression is a useful marker for lung lesions such as pulmonary edema [14,15]. The Aqp1 and Aqp5 expressions are known to be altered by radiation [16,17]. Thus, the present study investigated the mRNA expression levels of these marker genes as well as the pathology to assess the biological responses from inhaled ^56^MnO_2_ particles. Recently, gene expression markers have been extensively investigated for radiation dosimetry purposes. Studies have demonstrated that gene expression of cell cycle-related or apoptosis-related genes, such as Ccng1 and Bax, was suitable for bio-dosimetry [18,19]. Additionally, we identified these gene expressions in the lung to confirm the radiation responses.

## 2. Materials and Methods

### 2.1. Animals

Specific pathogen-free male C57BL mice (7 weeks old) were purchased from the Kazakh Scientific Center of Quarantine and Zoonotic Diseases, Almaty, Kazakhstan. Animals were housed individually in plastic cages (one mouse/cage) and maintained with free access to the basal diet and tap water. The room was maintained at 19–22 °C with a relative humidity of 30–70% and a 12-h light cycle. The animal facility was of the conventional type. Mice were randomly divided into 6 groups at 10 weeks old: Mn56 × 0.3 (*n* = 19), Mn56 × 1 (*n* = 19), Mn56 × 3 (*n* = 19), Cold-Mn (*n* = 16), Co60 (*n* = 16), and Control (*n* = 16). Body weights were measured every week afterward. The Mn56 × 0.3, Mn56 × 1, and Mn56 × 3 groups were exposed to 3 different activities of ^56^MnO_2_ particles (100 mg) of 8 × 10^7^, 2.4 × 10^8^, and 8 × 10^8^ Bq, respectively. Mice were placed in an exposure box, and ^56^MnO_2_ particles were air-pressure sprayed, as described previously [5]. After 1 h, the animals were moved to new cages to stop the exposure. The Cold-Mn group was exposed to nonradioactive MnO_2_ particles (100 mg) for 1 h. Three mice from each Mn56 group were necropsied to assess the absorbed dose at 0.5 h post exposure. The Co60 group received 2 Gy of external ^60^Co γ-ray whole-body irradiation. All animal groups were brought to the ^56^Mn exposure facility to maintain the same environment. Mice were necropsied on post-exposure days 3, 14, and 70 between 11:00 and 17:00, 5 mice/group on days 3 and 14, and 6 mice/group on day 70. The mice were sacrificed by removing their entire blood from an abdominal aorta under anesthesia with isoflurane (Fujifilm Wako Pure Chemical Co., Tokyo, Japan). The thymus, spleen, lung, heart, liver, kidney, and testis were dissected, and weighed. The lung was stored in RNA Save solution (Biological Industries Ltd., Beit Alfa, Israel) for RNA extraction, and part of it was fixed in 10% formalin. The animal study was approved by the Animal Experiment Ethics Committee of Semey Medical University, Semey, Kazakhstan (document #3-30.11.2018). The study was performed in compliance with the ARRIVE guidelines.

### 2.2. Irradiation and Dosimetry

Details of irradiation using ^56^MnO_2_ particles and the internal dose estimation have been previously described [5]. Briefly, MnO_2_ particles (Rare Metallic Co., Tokyo, Japan, particle diameters range 1–19 µm) were radio-activated by a neutron beam in the Baikal-1 nuclear reactor at the National Nuclear Center, Kurchatov, Kazakhstan. The ^56^MnO_2_ particles were then air-pressure sprayed into a sealed exposure box containing 19 mice. The animals were moved to new cages after 1 h of exposure. Three mice per group were euthanized 30 min later. The radioactivity of pieces of each organ was measured with a γ-spectrometer. Absorbed fractions of energy from β and γ-irradiation of ^56^Mn in organs were calculated using the Monte Carlo code (version MCNP-4C) and the mathematical phantom of the mouse. A ^60^Co radiotherapy machine, Teragam K-2 unit (UJP Praha, Praha-Zbraslav, Czech Republic), was used for whole-body γ-ray irradiation of 2.0 Gy (1.0 Gy/min).

### 2.3. Pathology

Formalin-fixed tissues were embedded in paraffin. Sections of 4-µm thickness were prepared and stained with HE.

### 2.4. Measurement of mRNA Levels by Quantitative Reverse Transcription Polymerase Chain Reaction (RT-PCR)

An RNA extraction solution, Isogen II (Nippon Gene Co., Tokyo, Japan), was used to isolate total RNA from tissue pieces of the lung stored in RNA Save solution. The cDNA was synthesized by incubating 2–4 µg of total RNA with 100 U of ReverTra Ace reverse transcriptase (Toyobo Co., Osaka, Japan) with a mixture of 20 pmol random hexamers pdN6 and 5 pmol oligo-dT(15) primers (Takara Bio Inc., Kusatsu, Japan). A qPCR instrument, StepOnePlus (Applied Biosystems/Life Technologies Co., Carlsbad, CA, USA), was employed for cDNA measurement with Thunderbird Next Sybr qPCR Mix Kit (Toyobo Co.). PCR fragments for each cDNA were prepared separately and purified by gel electrophoresis before quantitative analysis. The DNA sequences were confirmed by Fasmac Co., Ltd. (Atsugi, Japan). The extracted DNA fragments were used as standards for quantification. PCR conditions were 2 min of initial denaturation followed by 40 cycles of 5 s at 95 °C and 35 s at 60 °C. The measured mRNA levels were normalized with reference to the β-actin mRNA levels. Specific primer sets for genes are listed in Table 1.

### 2.5. Statistical Analysis

All values are expressed as mean ± standard error of the mean. Dunnett’s test was performed to compare the mRNA levels between the three doses of Mn56 groups and the cold Mn or between the Co60 group and the control group. The R package ‘SimComp’ was used for calculation (http://cran.r-project.org, accessed on 3 March 2023). The individual data and the calculated *p*-values of statistical comparisons are listed in supplementary files (Tables S1–S3).

## 3. Results

### 3.1. Estimated Doses of Internal Irradiation in the Lung

The estimation of the accumulated doses in the mice that are internally exposed to ^56^MnO_2_ was previously published [3]. The absorbed doses in the lung were 25 ± 5, 96 ± 13, and 250 ± 50 mGy for the Mn56 × 0.3, Mn56 × 1, and Mn56 × 3 groups, respectively, while the intestinal doses reached 250 ± 90, 910 ± 150, and 2300 ± 200 mGy, respectively.

### 3.2. Body Weight Changes

**Figure 1:**
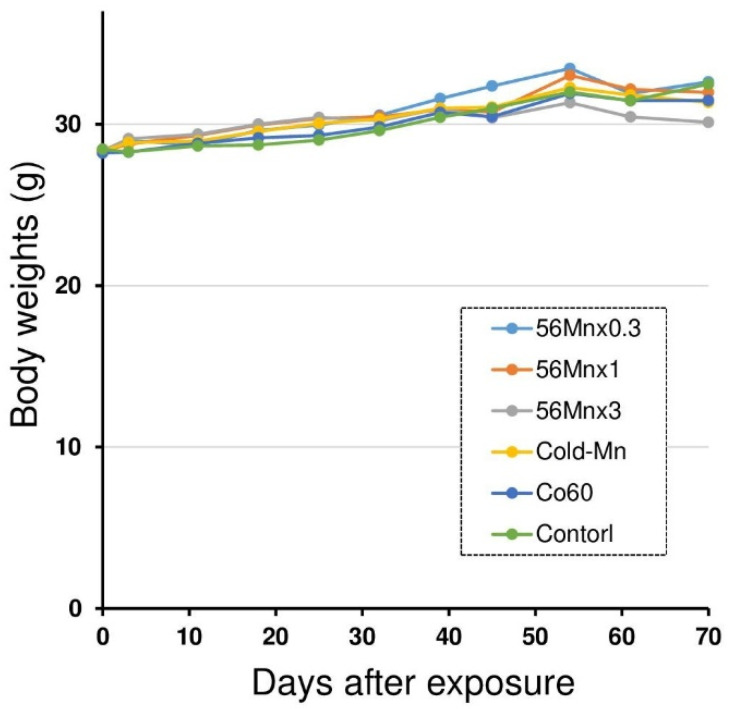
Changes in body weight after exposure.

Post-exposure body weight changes are shown in Figure 1. The initial average body weight was 28.4 ± 0.2 g. The body weights steadily increased in every group post exposure. No significant differences were found in the average body weights among the groups during the experiment.

Male C57BL mice were exposed to three different activities of ^56^MnO_2_ particles (Mn56 × 0.3, Mn56 × 1, or Mn56 × 3), nonradioactive MnO_2_ particles (Cold-Mn), 2 Gy of whole-body irradiation of ^60^Co γ-ray (Co60), or Control. The body weights steadily increased in every group after the exposure without significant differences among the groups during the study.

### 3.3. Organ Weights

Relative weights of the thymus, spleen, lung, heart, liver, kidney, and testis on days 3, 14, and 70 post exposure are summarized in Table 2. No significant differences were found in organ weight among the groups during the experiment, except the thymus on day 3, which decreased in the Co60 group.

### 3.4. Histology of the Lung

**Figure 2:**
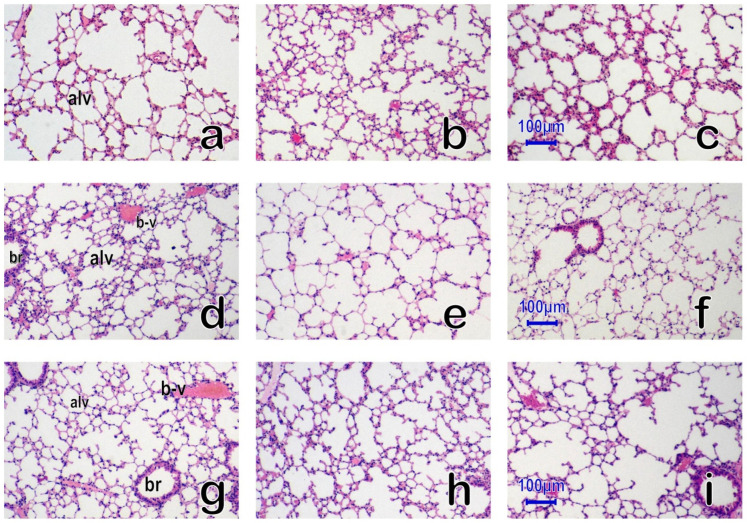
The mouse lungs on days 3 (**a**–**c**), 14 (**d**–**f**), and 70 (**g**–**i**) after ^56^MnO_2_ exposure (Mn56 × 3), 2 Gy of external γ-ray exposure (Co60), and Control, HE staining. No significant histological alternations were found in the lungs among the Mn56 × 3 (**a**,**d**,**g**), Co60 (**b**,**e**,**h**), and Control groups (**c**,**f**,**i**). The structure of the alveoli (alv) was normal, with the bronchiole (br) and blood vessels (b-v). Original magnification ×400. Bars indicate 100 µm.

Representative histology of the lung with hematoxylin and eosin (HE) staining on days 3, 14, and 70 post exposure in the Mn56 × 3, Co60, and the Control groups is shown in Figure 2. No remarkable changes were observed in the lungs among the groups. The structure of the alveoli was typical, with no signs of alveolar wall thickening.

### 3.5. Expression of Biodosimetry Marker Genes, Ccng1, and Bax, in the Lung on Day 3

**Figure 3:**
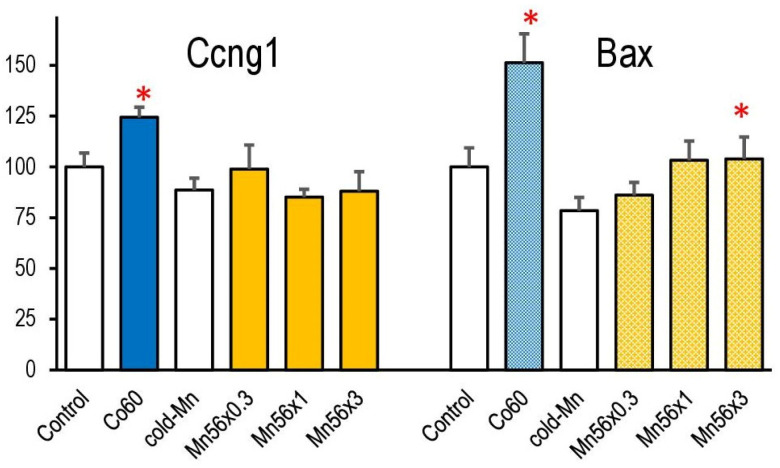
Relative mRNA expression levels of Ccng1 and Bax in the lung in mice on day 3 post exposure to ^56^MnO_2_ particles (Mn56 × 1, Mn56 × 2, and Mn56 × 3), nonradioactive MnO_2_ particles (Cold-Mn), or 2 Gy of external γ-rays (Co60). * *p* < 0.05 vs. Control or Cold-Mn. Bax expression increased in Co60 and Mn56 × 3 groups, while ccng1 expression increased only in the Co60 group.

The mRNA levels of both Ccng1 and Bax in the lung significantly increased in the Co60 group on day 3. Only the Bax expression was significantly increased in the Mn56 × 3 group (Figure 3).

### 3.6. Gene Expressions of Aqp1, Aqp5, and Smad7 in the Lung

**Figure 4:**
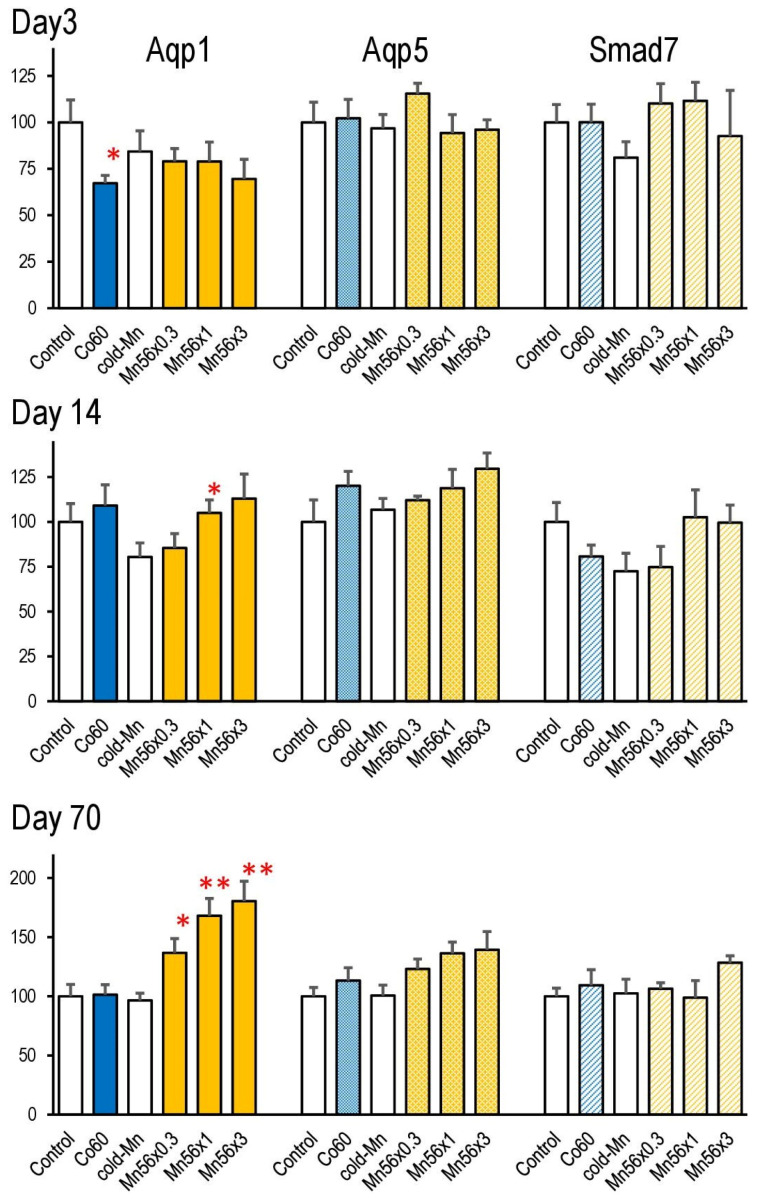
Relative mRNA expression levels of Aqp1, Aqp5, and Smad7 in the lung on days 3, 14, and 70 post exposure to ^56^MnO_2_ particles (Mn56 × 0.3, Mn56 × 1, and Mn56 × 3), nonradioactive MnO_2_ particles (Cold Mn), or 2Gy of external γ-rays (Co60). ** *p* < 0.01, * *p* < 0.05 vs. Control or Cold-Mn. Aqp1 expression started to increase on day 14, then significantly up-regulated in all Mn56 groups on day 71.

Figure 4 presents the mRNA expression of Aqp1, Aqp5, and Smad7 in each group on post-exposure days 3, 14, and 70. Aqp1 mRNA expression significantly decreased only in the Co60 group on day 3, whereas it increased in the Mn56 groups on days 14 and 70. No significant changes were noted in the Aqp5 expression among the groups. No changes were found in Smad7 mRNA expression.

## 4. Discussion

The role of the residual radioactivity produced by the bomb must be explored to better understand the biological impacts of the atomic bombing [1,2]. The neutron-activated radionuclides in the soil of the hypocenter areas at Hiroshima and Nagasaki include ^24^Na, ^28^Al, ^31^Si, ^32^P, ^56^Mn, and others [20]. Interestingly, studies of the early entrants showed that ‘risks’ were higher among persons who entered the city on the day of the bombing than those who entered later, which suggested that the corresponding radioisotopes may be short-lived [1,2]. Since ^56^Mn (t_1/2_ = 2.48 h) is one of the dominant radioactive isotopes during the first day after the atomic bomb explosion, it was chosen as a representative nucleus. Our previous studies in rats showed that exposure to ^56^Mn significantly impacted the lungs’ gene/protein expression [7]. We investigated mice for the first time to evaluate the effects of ^56^MnO_2_ particles further. The present study demonstrated that ^56^MnO_2_ exposure caused late increases in the Aqp1 mRNA expression in the lung at doses of <0.25 Gy.

We previously developed an exposure system for radioactive particles in laboratory animals to investigate the effects of ^56^MnO_2_ [21]. We exposed mice to ^56^MnO_2_ particles for the first time using this system. The same amount of ^56^MnO_2_, with the same specific activity (2.7 × 10^8^ Bq/100 mg) as our previous rat studies, was used in the Mn56 × 1 group. The estimated absorbed dose in each organ was approximately three times higher than that in rats due to the smaller body size in mice [5]. Body weights steadily increased in all groups during the experiment, indicating that the exposure did not severely influence the animal’s health. The thymus weight reduction, which is the classical radiation exposure indicator, was only found in the Co60 group, which confirmed the organ-dependent distribution of ^56^MnO_2_ [22].

Recently, gene expression changes have been recognized as radiation exposure markers [18,19]. These marker genes include cell cycle-related Ccng1, Cdk1na, apoptosis-related Bax, and Fas genes [23,24,25]. Many gene expression studies have focused on developing bio-dosimetry methods using peripheral blood samples. Additionally, gene expression changes after irradiation are found in various organs and are also useful predictors for tissue-dependent radiation effects [26]. We confirmed that the mRNA expression of radiation-sensitive genes, Ccng1, and Bax, was elevated by 2 Gy of external γ-irradiation on day 3. We found that ^56^MnO_2_ exposure only increased Bax gene expression, which coincided with the lung receiving lower doses of radiation (<0.25 Gy as calculated).

Generally, the lung is classified as radiation-resistant because of its relatively low mitotic activity [8]. Many studies have demonstrated that the lung resists low-dose radiation [27,28]. Thus, it may not be surprising that irradiation at less than 2 Gy did not induce morphological changes earlier than 70 days post exposure in the present study. However, high doses can cause radiation injuries. High doses of external radiation exposure to the lungs can induce radiation pneumonitis and fibrosis in animals and humans [8,9,11]. These radiation-induced lung injuries are major complications in radiotherapy and, therefore, have been extensively investigated [29]. The transcriptome analysis has found an essential role of TGF-β in this lung lesion. A mouse study revealed a long-term elevated TGF-β mRNA expression in the lung after thoracic irradiation [30]. TGFβ physiologically regulates wound healing and subsequent fibrosis. However, this process often leads to chronic pathological results, as found in the irradiated lung [31]. TGF-β binds to its cell surface receptor to activate the Smad signaling pathway, leading to gene transcription related to diverse biological processes, including fibrosis [32]. Irradiation could change the expression of the Smad genes, Smad 2 and Smad7 [13]. Our previous study in rats revealed significantly increased Smad7 mRNA levels due to ^56^MnO_2_ on day 3 post exposure, which may be related to the physiological healing processes of lung tissue damage [7]. However, Smad7 expression was not affected by ^56^MnO_2_ or γ-ray exposure in the mouse. Neither TGF-β or Smad3 expression was altered.

Other expression gene markers related to lung injury were water-selective channel proteins, AQPs. They are ubiquitously expressed membrane proteins that control the plasma membrane’s water permeability [14]. AQP1 is localized in sub-epithelial connective tissues and capillaries in the lung, whereas AQP5 is found in the epithelium [33]. These two types of AQPs provide a major means of water movement in the peripheral lung [34]. The expression changes in Aqp mRNAs after radiation or other lung injury-inducing chemicals have been well-documented [15]. These changes are parallel with pathophysiological alterations in the lung, although expression changes in Aqp differ among experimental models or species. The Aqp1 gene expression increased in the pulmonary edema models, while the Aqp1 and Aqp5 expression decreased in acute lung injury models [35,36]. The present study revealed that 2 Gy of external γ-rays suppressed Aqp1 mRNA expression on day 3 post exposure, which recovered afterward. However, ^56^MnO_2_ exposure brought ‘a late effect’ on the Aqp1 expression. The level of Aqp1 mRNA started to elevate on day 14 post exposure and significantly increased in all Mn56 groups on day 70. This might indicate pathophysiological alternation without apparent histological changes. However, our previous study in rats revealed no changes in Aqp expression later (on day 62 post exposure) [7], which is probably due to the difference in the radiation doses between the two studies. The absorbed radiation doses in the rat lung ranged from 25 to 75 mGy, while they reached 250 mGy in the present study. The effects on gene expression of 2 Gy of external irradiation were only short-term in any case. Conversely, the internal exposure to ^56^Mn appeared to have higher biological impacts on the lung tissue, which may be explained by the peculiarities of spatial dose distributions in the biological tissue from beta-particle radiation from inhaled ^56^Mn micro-particles [37,38]. Although the protein levels of Aqp1 were not determined in the present study, previous investigations revealed protein expressions of Aqp1 and Aqp5 comparable to their mRNA expression levels during lung injury [39,40].

The behavior of radionuclides following internal exposure depends on their physical and chemical form [41]. Soluble radioactive chemicals are easily absorbed into the bloodstream and distributed to all tissues, giving an equivalent radiation dose when animals are exposed. However, radiation doses vary among organs, as it is delivered to the skin, the lungs, and eventually, the gastrointestinal tract if the radionuclides are insoluble, such as ^56^MnO_2_. Irradiation from these insoluble particles may be less hazardous in terms of carcinogenesis than uniformly distributed radionuclides with the same activity. However, these insoluble radioactive particles could have higher biological impacts on specific target organs, as our present study indicated.

Mn is a neurotoxic chemical at high doses [42]. Experimental studies have suggested that Mn exposure leads to a primary inflammatory reaction in the respiratory tract [43,44]. In the present study, no signs of pulmonary inflammation were noted in the cold-Mn group, indicating that the exposed MnO_2_ level was below the toxic. The estimated level of MnO_2_ based on the radioactivity at 0.5 h after the end of exposure was about 26 µg/g lung tissue.

Our findings demonstrated that ^56^MnO_2_ particle exposure produced more prominent biological responses than an external γ-irradiation in mice as well as rats, although the radiation sensitivity of the lung differs between the two species, as discussed above. These results suggest the significant roles of internal exposure to residual radioactive particles, although the quantitative relevance of our results on the human health effects of residual radiation after the atomic bombing is not conclusive because of their unknown absorbed radiation doses.

## 5. Conclusions

^56^Mn is one of the dominant sources of the residual radiation produced in the soil soon after an atomic bomb explosion. We investigated the effect of internal exposure to ^56^MnO_2_ particles on the lungs of male C57BL mice by examining the pathology and gene expression. Inhalation exposure to ^56^MnO_2_ particles resulted in absorbed doses of 26–250 mGy in the lung and caused significant impacts on gene expressions of Aqp1 on post-exposure days 14 and 70. These changes were not observed in external γ-irradiation of 2 Gy, suggesting that ^56^MnO_2_ particle exposure has significantly higher biological effects than external irradiation on the lung.

## Figures and Tables

**Table 1:** Q-PCR primers.

Gene	GenBank Accession#	Q-PCR Primer Sequences (5′ -> 3′)	
		Forward	Reverse
Ccng1	NM_009831	CCTGCCACTGCAGGATCATA	AAGGTCAAATCTCGGCCACTT
Bax	NM_007527	CGTGGACACGGACTCCCCCC	TGATCAGCTCGGGCACTTTA
Aqp1	NM_007472	ACCTGCTGGCGATTGACTACA	CATAGATGAGCACTGCCAGGG
Aqp5	NM_009701	CTCCCCAGCCTTATCCATTG	CACGATCGGTCCTACCCAGA
Smad7	AF015260	TTGCTGTGAATCTTACGGGAAG	GGTTTGAGAAAATCCATTGGGT
Actb	NM_007393.5	CTGTCCCTGTATGCCTCTGGTC	TGAGGTAGTCCGTCAGGTCCC

**Table 2:** Relative organ weights in mice were exposed to ^56^MnO_2_ (Mn56 × 0.3, Mn56 × 1, and Mn56 × 3), nonradioactive MnO_2_ (Cold-Mn), and external γ-rays (Co60). The thymus weight decreased on day 3 only in the Co60 group. Otherwise, there were no significant differences in any organ weights.

	Body Weights (g)	Thymus (g/kg bw)	Spleen (g/kg bw)	Lung (g/kg bw)	Heart (g/kg bw)	Liver (g/kg bw)	Kidney (g/kg bw)	Testis (g/kg bw)
Day 3								
Mn56 × 0.3	28.9 ± 1.44	1.5 ± 0.04	4 ± 0.56	10.4 ± 0.87	5.8 ± 0.19	52 ± 3.0	15 ± 0.55	6.6 ± 0.92
Mn56 × 1	28.4 ± 1.18	1.4 ± 0.23	3.6 ± 0.2	9.2 ± 0.62	6.5 ± 0.33	52 ± 1.7	16 ± 0.71	7.1 ± 0.57
Mn56 × 3	28.3 ± 1.33	1.6 ± 0.14	4.3 ± 0.59	9.7 ± 0.27	6 ± 0.29	50 ± 2.1	16 ± 0.60	6.2 ± 0.53
Co60	27.9 ± 1.08	1.0 ± 0.13 *	3.2 ± 0.6	10.2 ± 0.69	5.9 ± 0.24	51 ± 2.8	15 ± 0.45	7.4 ± 0.64
Cold-Mn	28.1 ± 1.3	1.5 ± 0.08	4.1 ± 0.48	10.6 ± 0.38	6.2 ± 0.22	53 ± 1.8	16 ± 0.66	7.3 ± 0.66
Control	27.8 ± 0.86	1.4 ± 0.15	3.8 ± 0.38	9.7 ± 0.4	6.1 ± 0.27	52 ± 1.7	15 ± 0.27	7.1 ± 0.69
								
Day 14								
Mn56 × 0.3	28.5 ± 1.04	1.5 ± 0.15	3.2 ± 0.25	8.9 ± 0.40	5.5 ± 0.14	51 ± 2.7	15 ± 0.35	7.5 ± 0.51
Mn56 × 1	29.4 ± 0.75	1.8 ± 0.1	3.7 ± 0.18	9.2 ± 0.76	5.9 ± 0.18	52 ± 1.6	16 ± 0.96	5.6 ± 1.1
Mn56 × 3	28.9 ± 1.02	2.1 ± 0.29	4.1 ± 0.35	9.7 ± 0.49	6.0 ± 0.45	47 ± 4.6	16 ± 0.32	7.4 ± 0.68
Co60	29.4 ± 0.63	1.2 ± 0.22	2.8 ± 0.23	9.5 ± 0.51	5.6 ± 0.22	48 ± 1.9	17 ± 0.34	5.7 ± 0.32
Cold-Mn	28.9 ± 0.89	1.7 ± 0.17	3.9 ± 0.29	9 ± 0.49	5.5 ± 0.28	53 ± 2.5	16 ± 0.44	7.8 ± 0.83
Control	29 ± 0.94	1.6 ± 0.14	3.2 ± 0.10	9.9 ± 0.48	5.6 ± 0.1	49 ± 1.4	17 ± 1.02	6.8 ± 0.56
								
Day 70								
Mn56 × 0.3	32.0 ± 0.96	1.4 ± 0.14	2.9 ± 0.19	9.3 ± 0.30	5.4 ± 0.19	45 ± 1.0	14 ± 0.46	7.8 ± 0.74
Mn56 × 1	32.6 ± 1.36	1.3 ± 0.31	3.3 ± 0.41	9.0 ± 0.65	5.5 ± 0.25	47 ± 1.3	15 ± 0.68	6.5 ± 0.39
Mn56 × 3	30.1 ± 0.80	1.4 ± 0.12	3.4 ± 0.42	9.0 ± 0.59	5.9 ± 0.17	46 ± 2.6	15 ± 0.50	7.3 ± 0.90
Co60	31.5 ± 1.16	1.3 ± 0.10	2.7 ± 0.22	8.0 ± 0.18	5.5 ± 0.18	49 ± 2.0	16 ± 0.34	6.5 ± 0.20
Cold-Mn	31.4 ± 1.14	1.4 ± 0.12	3.2 ± 0.37	8.8 ± 0.19	6.1 ± 0.27	45 ± 0.8	16 ± 0.15	7.4 ± 0.65
Control	32.5 ± 1.62	1.1 ± 0.11	2.7 ± 0.27	8.9 ± 0.21	5.4 ± 0.28	47 ± 0.8	16 ± 1.15	6.8 ± 0.37

* *p* < 0.05 vs. Control.

## Data Availability

Individual body/organ weights and Q-PCR data are presented in Supplementary Materials.

## Supplementary Materials

The following supporting information can be downloaded at: https://www.mdpi.com/article/10.3390/cimb45040209/s1, Table S1: Organ Weight Data; Table S2: QPCR Data1; Table S3: QPCR Data2.
