# Supplementary material for: Effects of Internal Exposure of Radioactive 56MnO2 Particles on the Lung in C57BL Mice
Source: Curr Issues Mol Biol. 2023 Apr 6;45(4):3208–18. doi: 10.3390/cimb45040209 (PMC10137078; doi:10.3390/cimb45040209)
Supplement: Supplementary file 1 [file cimb-45-00209-s001.zip › TableS1 Organ Weight Data.pdf]

Body and Organ Weights - For Table 1

Day3

	Rat#	B.W.	Organ weights Thymus	Lung	Heart	Liver	spleen	Kidney	Testis	Organ relative weights Thymus	Lung	Heart	Liver	spleen	Kidney	Testis
Mn56x0.3-3D	1-01	24.9	0.04	0.29	0.15	1.38	0.12	0.35	0.21	1.6	11.6	6.0	55.4	4.8	14.1	8.4
	1-02	26.9	0.04	0.32	0.17	1.11	0.13	0.4	0.24	1.5	11.9	6.3	41.3	4.8	14.9	8.9
	1-03	28.4	0.04	0.27	0.15	1.67	0.14	0.44	0.14	1.4	9.5	5.3	58.8	4.9	15.5	4.9
	1-04	31.5	0.05	0.36	0.19	1.56	0.1	0.54	0.2	1.6	11.4	6.0	49.5	3.2	17.1	6.3
	1-05	32.7	0.05	0.24	0.18	1.71	0.07	0.47	0.14	1.5	7.3	5.5	52.3	2.1	14.4	4.3
		28.9								1.5	10.4	5.8	51.5	4.0	15.2	6.6
		1.44								0.04	0.87	0.19	2.98	0.56	0.55	0.92
Mn56x1-3D	2-01	24.6	0.04	0.2	0.17	1.35	0.09	0.4	0.18	1.6	8.1	6.9	54.9	3.7	16.3	7.3
	2-02	27	0.02	0.29	0.15	1.55	0.08	0.39	0.2	0.7	10.7	5.6	57.4	3.0	14.4	7.4
	2-03	28.8	0.05	0.31	0.17	1.62	0.11	0.42	0.17	1.7	10.8	5.9	56.3	3.8	14.6	5.9
	2-04	30.3	0.03	0.25	0.2	1.45	0.11	0.46	0.18	1.0	8.3	6.6	47.9	3.6	15.2	5.9
	2-05	31.2	0.06	0.26	0.23	1.63	0.13	0.57	0.28	1.9	8.3	7.4	52.2	4.2	18.3	9.0
		28.4								1.4	9.2	6.5	53.7	3.6	15.7	7.1
		1.18								0.23	0.62	0.33	1.70	0.20	0.71	0.57
Mn56x3-3D	3-01	24.8	0.04	0.24	0.13	1.31	0.08	0.34	0.13	1.6	9.7	5.2	52.8	3.2	13.7	5.2
	3-02	26.1	0.04	0.28	0.16	1.22	0.17	0.43	0.21	1.5	10.7	6.1	46.7	6.5	16.5	8.0
	3-03	28.1	0.06	0.26	0.16	1.57	0.12	0.46	0.17	2.1	9.3	5.7	55.9	4.3	16.4	6.0
	3-04	31.2	0.04	0.29	0.19	1.43	0.12	0.54	0.2	1.3	9.3	6.1	45.8	3.8	17.3	6.4
	3-05	31.4	0.05	0.3	0.22	1.45	0.11	0.5	0.16	1.6	9.6	7.0	46.2	3.5	15.9	5.1
		28.3								1.6	9.7	6.0	49.5	4.3	16.0	6.2
		1.33								0.14	0.27	0.29	2.05	0.59	0.60	0.53
Co60-3D	4-01	24.4	0.02	0.27	0.14	1.16	0.08	0.35	0.19	0.8	11.1	5.7	47.5	3.3	14.3	7.8
	4-02	26.9	0.03	0.24	0.15	1.46	0.06	0.36	0.18	1.1	8.9	5.6	54.3	2.2	13.4	6.7
	4-03	28.4	0.04	0.23	0.17	1.7	0.08	0.45	0.15	1.4	8.1	6.0	59.9	2.8	15.8	5.3
	4-04	29.1	0.03	0.33	0.16	1.28	0.16	0.44	0.23	1.0	11.3	5.5	44.0	5.5	15.1	7.9
	4-05	30.8	0.02	0.35	0.21	1.48	0.07	0.48	0.28	0.6	11.4	6.8	48.1	2.3	15.6	9.1
		27.9								1.0	10.2	5.9	50.7	3.2	14.9	7.4
		1.08								0.13	0.69	0.24	2.82	0.60	0.45	0.64
coldMn-3D	5-01	24	0.03	0.24	0.16	1.22	0.06	0.36	0.18	1.3	10.0	6.7	50.8	2.5	15.0	7.5
	5-02	26.8	0.04	0.28	0.15	1.41	0.11	0.41	0.13	1.5	10.4	5.6	52.6	4.1	15.3	4.9
	5-03	28.6	0.04	0.28	0.19	1.51	0.13	0.51	0.22	1.4	9.8	6.6	52.8	4.5	17.8	7.7
	5-04	29.3	0.05	0.32	0.17	1.75	0.16	0.49	0.23	1.7	10.9	5.8	59.7	5.5	16.7	7.8
	5-05	31.8	0.05	0.38	0.2	1.57	0.13	0.45	0.28	1.6	11.9	6.3	49.4	4.1	14.2	8.8
		28.1								1.5	10.6	6.2	53.1	4.1	15.8	7.3
		1.30								0.08	0.38	0.22	1.78	0.48	0.66	0.66
C-3D	6-01	24.8	0.03	0.26	0.16	1.34	0.08	0.38	0.14	1.2	10.5	6.5	54.0	3.2	15.3	5.6
	6-02	27.1	0.05	0.23	0.16	1.41	0.1	0.39	0.15	1.8	8.5	5.9	52.0	3.7	14.4	5.5
	6-03	29	0.03	0.28	0.15	1.61	0.08	0.44	0.23	1.0	9.7	5.2	55.5	2.8	15.2	7.9
	6-04	29.7	0.05	0.27	0.19	1.36	0.14	0.42	0.21	1.7	9.1	6.4	45.8	4.7	14.1	7.1
	6-05	28.3	0.04	0.3	0.19	1.46	0.13	0.44	0.26	1.4	10.6	6.7	51.6	4.6	15.5	9.2
		27.8								1.4	9.7	6.1	51.8	3.8	14.9	7.1
		0.86								0.15	0.40	0.27	1.66	0.38	0.27	0.69

Day14

	Rat#	B.W.	Organ weights Thymus	Lung	Heart	Liver	spleen	Kidney	Testis	Organ relative weights Thymus	Lung	Heart	Liver	spleen	Kidney	Testis
Mn56x0.3-14D	1-06	25.2	0.05	0.24	0.14	1.03	0.08	0.35	0.19	2.0	9.5	5.6	40.9	3.2	13.9	7.5
	1-07	28	0.03	0.28	0.16	1.51	0.09	0.44	0.16	1.1	10.0	5.7	53.9	3.2	15.7	5.7
	1-08	28	0.04	0.25	0.14	1.49	0.1	0.41	0.25	1.4	8.9	5.0	53.2	3.6	14.6	8.9
	1-09	30	0.05	0.23	0.16	1.53	0.07	0.43	0.23	1.7	7.7	5.3	51.0	2.3	14.3	7.7
	1-10	31.3	0.05	0.27	0.18	1.78	0.12	0.43	0.24	1.6	8.6	5.8	56.9	3.8	13.7	7.7
		28.5								1.5	8.9	5.5	51.2	3.2	14.5	7.5
		1.04								0.15	0.40	0.14	2.74	0.25	0.35	0.51
Mn56x1-14D	2-06	26.8	0.04	0.25	0.16	1.46	0.1	0.37	0.16	1.5	9.3	6.0	54.5	3.7	13.8	6.0
	2-07	29.4	0.05	0.29	0.16	1.35	0.09	0.42	0.16	1.7	9.9	5.4	45.9	3.1	14.3	5.4
	2-08	29.1	0.06	0.24	0.18	1.55	0.11	0.47	0.19	2.1	8.2	6.2	53.3	3.8	16.2	6.5
	2-09	30.3	0.06	0.35	0.17	1.58	0.11	0.45	0.25	2.0	11.6	5.6	52.1	3.6	14.9	8.3
	2-10	31.3	0.06	0.22	0.2	1.7	0.13	0.6	0.05	1.9	7.0	6.4	54.3	4.2	19.2	1.6
		29.4								1.8	9.2	5.9	52.0	3.7	15.7	5.6
		0.75								0.10	0.76	0.18	1.58	0.18	0.96	1.10
Mn56x3-14D	3-06	25.3	0.05	0.26	0.13	1.35	0.09	0.41	0.23	2.0	10.3	5.1	53.4	3.6	16.2	9.1
	3-07	29.2	0.09	0.32	0.17	0.84	0.15	0.5	0.23	3.1	11.0	5.8	28.8	5.1	17.1	7.9
	3-08	28.7	0.05	0.24	0.17	1.47	0.1	0.46	0.23	1.7	8.4	5.9	51.2	3.5	16.0	8.0
	3-09	29.8	0.04	0.26	0.23	1.41	0.14	0.45	0.2	1.3	8.7	7.7	47.3	4.7	15.1	6.7
	3-10	31.5	0.07	0.32	0.17	1.65	0.11	0.5	0.16	2.2	10.2	5.4	52.4	3.5	15.9	5.1
		28.9								2.1	9.7	6.0	46.6	4.1	16.1	7.4
		1.02								0.29	0.49	0.45	4.58	0.35	0.32	0.68
Co60-14D	4-06	28.2	0.05	0.25	0.16	1.48	0.1	0.49	0.16	1.8	8.9	5.7	52.5	3.5	17.4	5.7
	4-07	28.7	0.03	0.25	0.15	1.25	0.07	0.49	0.2	1.0	8.7	5.2	43.6	2.4	17.1	7.0
	4-08	29.6	0.02	0.34	0.15	1.3	0.09	0.5	0.16	0.7	11.5	5.1	43.9	3.0	16.9	5.4

	4-09	28.9	0.05	0.27	0.18	1.49	0.08	0.48	0.15	1.7	9.3	6.2	51.6	2.8	16.6	5.2
	4-10	31.8	0.03	0.29	0.19	1.54	0.07	0.49	0.17	0.9	9.1	6.0	48.4	2.2	15.4	5.3
		29.4								1.2	9.5	5.6	48.0	2.8	16.7	5.7
		0.63								0.22	0.51	0.22	1.86	0.23	0.34	0.32
coldMn-14D	5-06	27.8	0.06	0.2	0.18	1.33	0.12	0.45	0.22	2.2	7.2	6.5	47.8	4.3	16.2	7.9
	5-07	26.7	0.05	0.25	0.14	1.26	0.08	0.41	0.26	1.9	9.4	5.2	47.2	3.0	15.4	9.7
	5-08	28.7	0.05	0.29	0.16	1.73	0.13	0.48	0.21	1.7	10.1	5.6	60.3	4.5	16.7	7.3
	5-09	29.2	0.04	0.26	0.14	1.56	0.1	0.43	0.27	1.4	8.9	4.8	53.4	3.4	14.7	9.2
	5-10	32	0.04	0.3	0.18	1.8	0.13	0.46	0.16	1.3	9.4	5.6	56.3	4.1	14.4	5.0
		28.9								1.7	9.0	5.5	53.0	3.9	15.5	7.8
		0.89								0.17	0.49	0.28	2.49	0.29	0.44	0.83
C-14D	6-06	26.8	0.04	0.27	0.15	1.39	0.09	0.41	0.16	1.5	10.1	5.6	51.9	3.4	15.3	6.0
	6-07	28.8	0.06	0.23	0.17	1.41	0.1	0.56	0.19	2.1	8.0	5.9	49.0	3.5	19.4	6.6
	6-08	27.4	0.04	0.29	0.15	1.26	0.08	0.4	0.15	1.5	10.6	5.5	46.0	2.9	14.6	5.5
	6-09	29.8	0.04	0.3	0.17	1.4	0.1	0.58	0.26	1.3	10.1	5.7	47.0	3.4	19.5	8.7
	6-10	32.1	0.06	0.34	0.17	1.7	0.1	0.53	0.23	1.9	10.6	5.3	53.0	3.1	16.5	7.2
		29.0								1.6	9.9	5.6	49.3	3.2	17.1	6.8
		0.94								0.14	0.48	0.10	1.35	0.10	1.02	0.56

Day70

	Rat#	B.W.	Organ weights							Organ relative weights						
			Thymus	Lung	Heat	Liver	Spleen	Kidney	Testis	Thymus	Lung	Heart	Liver	spleen	Kidney	Testis
Mn56x0.3-70D	1-11	28.7	0.04	0.28	0.17	1.3	0.08	0.43	0.23	1.4	9.8	5.9	45.3	2.8	15.0	8.0
	1-12	31.0	0.06	0.28	0.17	1.41	0.09	0.43	0.21	1.9	9.0	5.5	45.5	2.9	13.9	6.8
	1-13	35.8	0.04	0.34	0.17	1.51	0.08	0.45	0.22	1.1	9.5	4.7	42.2	2.2	12.6	6.1
	1-14	32.8	0.05	0.26	0.19	1.63	0.09	0.51	0.22	1.5	7.9	5.8	49.7	2.7	15.5	6.7
	1-15	32.2	0.04	0.32	0.16	1.46	0.11	0.45	0.25	1.2	9.9	5.0	45.3	3.4	14.0	7.8
	1-16	31.3	0.03	0.3	0.17	1.39	0.11	0.48	0.35	1.0	9.6	5.4	44.4	3.5	15.3	11.2
		32.0								1.4	9.3	5.4	45.4	2.9	14.4	7.8
		0.96								0.14	0.30	0.19	1.00	0.19	0.46	0.74
Mn56x1-70D	2-11															
	2-12	29.5	0.02	0.25	0.17	1.34	0.09	0.42	0.18	0.7	8.5	5.8	45.4	3.1	14.2	6.1
	2-13	31.4	0.03	0.3	0.19	1.34	0.08	0.52	0.24	1.0	9.6	6.1	42.7	2.5	16.6	7.6
	2-14	31.6	0.05	0.21	0.17	1.52	0.08	0.41	0.2	1.6	6.6	5.4	48.1	2.5	13.0	6.3
	2-15	33.0	0.03	0.33	0.15	1.56	0.11	0.49	0.18	0.9	10.0	4.5	47.3	3.3	14.8	5.5
	2-16	37.6	0.09	0.38	0.21	1.88	0.18	0.62	0.27	2.4	10.1	5.6	50.0	4.8	16.5	7.2
		32.6								1.3	9.0	5.5	46.7	3.3	15.0	6.5
		1.36								0.31	0.65	0.25	1.25	0.41	0.68	0.39
Mn56x3-70D	3-11	28.2	0.03	0.26	0.18	1.34	0.07	0.43	0.16	1.1	9.2	6.4	47.5	2.5	15.2	5.7
	3-12	28.3	0.05	0.26	0.17	1.42	0.11	0.4	0.25	1.8	9.2	6.0	50.2	3.9	14.1	8.8
	3-13	31.4	0.05	0.26	0.19	1.09	0.1	0.41	0.16	1.6	8.3	6.1	34.7	3.2	13.1	5.1
	3-14	30.1	0.04	0.35	0.16	1.43	0.16	0.5	0.33	1.3	11.6	5.3	47.5	5.3	16.6	11.0
	3-15	29.4	0.05	0.23	0.16	1.54	0.08	0.45	0.19	1.7	7.8	5.4	52.4	2.7	15.3	6.5
	3-16	33.3	0.04	0.26	0.2	1.39	0.1	0.51	0.23	1.2	7.8	6.0	41.7	3.0	15.3	6.9
		30.1								1.4	9.0	5.9	45.7	3.4	14.9	7.3
		0.80								0.12	0.59	0.17	2.63	0.42	0.50	0.90
Co60-70D	4-11	29.5	0.05	0.23	0.15	1.47	0.07	0.48	0.21	1.7	7.8	5.1	49.8	2.4	16.3	7.1
	4-12	31.4	0.04	0.26	0.15	1.61	0.07	0.46	0.19	1.3	8.3	4.8	51.3	2.2	14.6	6.1
	4-13	28.5	0.04	0.24	0.17	1.46	0.1	0.47	0.2	1.4	8.4	6.0	51.2	3.5	16.5	7.0
	4-14	31.3	0.03	0.26	0.18	1.69	0.09	0.5	0.21	1.0	8.3	5.8	54.0	2.9	16.0	6.7
	4-15	31.4	0.04	0.24	0.18	1.38	0.1	0.46	0.2	1.3	7.6	5.7	43.9	3.2	14.6	6.4
	4-16	36.7	0.04	0.27	0.2	1.51	0.08	0.55	0.22	1.1	7.4	5.4	41.1	2.2	15.0	6.0
		31.5								1.3	8.0	5.5	48.6	2.7	15.5	6.5
		1.16								0.10	0.18	0.18	2.02	0.22	0.34	0.20
coldMn-70D	5-11	28.8	0.04	0.26	0.19	1.27	0.12	0.5	0.26	1.4	9.0	6.6	44.1	4.2	17.4	9.0
	5-12	32.1	0.06	0.27	0.2	1.46	0.14	0.51	0.22	1.9	8.4	6.2	45.5	4.4	15.9	6.9
	5-13	34.5	0.04	0.29	0.2	1.54	0.08	0.52	0.19	1.2	8.4	5.8	44.6	2.3	15.1	5.5
	5-14	28.4	0.04	0.27	0.2	1.38	0.09	0.51	0.27	1.4	9.5	7.0	48.6	3.2	18.0	9.5
	5-15	29.7	0.03	0.25	0.17	1.26	0.08	0.46	0.22	1.0	8.4	5.7	42.4	2.7	15.5	7.4
	5-16	34.6	0.05	0.31	0.18	1.54	0.08	0.47	0.21	1.4	9.0	5.2	44.5	2.3	13.6	6.1
		31.4								1.4	8.8	6.1	45.0	3.2	15.9	7.4
		1.14	0.00							0.12	0.19	0.27	0.84	0.37	0.65	0.65
C-70D	6-11	29.2	0.04	0.28	0.15	1.46	0.07	0.46	0.24	1.4	9.6	5.1	50.0	2.4	15.8	8.2
	6-12	31.8	0.04	0.26	0.17	1.45	0.08	0.55	0.21	1.3	8.2	5.3	45.6	2.5	17.3	6.6
	6-13	32.0	0.03	0.28	0.17	1.51	0.08	0.43	0.24	0.9	8.8	5.3	47.2	2.5	13.4	7.5
	6-14	31.0	0.03	0.28	0.2	1.37	0.06	0.45	0.19	1.0	9.0	6.5	44.2	1.9	14.5	6.1
	6-15	30.6	0.02	0.28	0.18	1.44	0.12	0.61	0.21	n.d.	9.2	5.9	47.1	3.9	19.9	6.9
	6-16	40.3	0.03	0.34	0.18	1.88	0.11	0.49	0.23	n.d.	8.4	4.5	46.7	2.7	12.2	5.7
		32.5								1.1	8.9	5.4	46.8	2.7	15.5	6.8
		1.62								0.11	0.21	0.28	0.79	0.27	1.15	0.37
