# Supplementary material for: Effects of Internal Exposure of Radioactive 56MnO2 Particles on the Lung in C57BL Mice
Source: Curr Issues Mol Biol. 2023 Apr 6;45(4):3208–18. doi: 10.3390/cimb45040209 (PMC10137078; doi:10.3390/cimb45040209)
Supplement: Supplementary file 1 [file cimb-45-00209-s001.zip › TableS2_QPCRdata1.pdf]

Lung-Qpcr data for Figure 3

Day 3	Rat#	Q-Pcr Bactin	Ccng1	x10/bac	M/SE	%M/SE	Bax	x10/bact	M/SE	%M/SE	Dunnett Test - p valu ccng1	Bax
Mn56x0.3-3D	1-01	22.8	24.39	10.7			1.06	0.46				
	1-02	26.7	31.98	12.0			1.91	0.71				
	1-03	27.9	32.46	11.6			1.70	0.61				
	1-04	23.7	43.57	18.4			1.65	0.70			vs coldMn	
	1-05	17.6	17.74	10.1	12.6	98.9	1.22	0.69	0.64	86.1	0.4647	0.2372
					1.50	11.8			0.05	6.3		
Mn56x1-3D	2-01	23.9	26.75	11.2			2.04	0.85				
	2-02	21.5	19.82	9.2			1.19	0.55				
	2-03	12.4	14.94	12.0			1.20	0.96				
	2-04	15.0	16.89	11.3			1.04	0.70				
	2-05	23.7	24.41	10.3	10.8	85.1	1.76	0.74	0.76	103.2	0.9155	0.0557
					0.48	3.8			0.07	9.5		
Mn56x3-3D	3-01	23.2	25.31	10.9			1.90	0.82				
	3-02	7.9	6.73	8.5			0.66	0.84				
	3-03	36.7	31.40	8.6			2.20	0.60				
	3-04	34.1	49.76	14.6			2.21	0.65				
	3-05	19.2	25.44	13.3	11.2	88.0	1.92	1.00	0.78	105.9	0.8048	0.045
					1.23	9.7			0.07	9.7		
Co60-3D	4-01	23.8	33.39	14.0			2.09	0.88				
	4-02	31.8	47.79	15.0			3.72	1.17				
	4-03	24.2	40.91	16.9			3.46	1.43				
	4-04	33.8	52.28	15.5			3.00	0.89			vs Control	
	4-05	13.7	23.93	17.5	15.8	124.4	1.66	1.22	1.12	151.3	0.0121	0.0119
					0.63	5.0			0.11	14.3		
coldMn-3D	5-01	20.0	23.31	11.7			1.04	0.52				
	5-02	31.5	29.58	9.4			1.70	0.54				
	5-03	39.5	38.05	9.6			1.81	0.46				
	5-04	20.3	26.28	12.9			1.32	0.65				
	5-05	31.4	39.61	12.6	11.2	88.6	1.90	0.60	0.55	75.1		
					0.74	5.8			0.03	4.5		
C-3D	6-01	34.9	39.53	11.3			2.23	0.64				
	6-02	27.8	40.25	14.5			1.99	0.72				
	6-03	31.1	40.03	12.9			2.89	0.93				
	6-04	27.2	39.71	14.6			2.33	0.86				
	6-05	22.3	22.70	10.2	12.7	100.0	1.23	0.55	0.74	100.0		
					0.87	6.9			0.07	9.4		

es
