# Supplementary material for: Effects of Internal Exposure of Radioactive 56MnO2 Particles on the Lung in C57BL Mice
Source: Curr Issues Mol Biol. 2023 Apr 6;45(4):3208–18. doi: 10.3390/cimb45040209 (PMC10137078; doi:10.3390/cimb45040209)
Supplement: Supplementary file 1 [file cimb-45-00209-s001.zip › TableS3_QPCRdata2.pdf]

Lung-Qpcr data for Figure 4

Day 3	Rat#	Q-Pcr Bactin	AQP1	x10/bact	M/SE	%M/SE	AQP5	x10/bact	M/SE	%M/SE	Smad7	x10/bact	M/SE	%M/SE	Dunnett Test - p values AQP1	AQP5	Smad7
Mn56x0.3-3D	1-01	22.8	1.57	0.69			4.89	2.15			1.86	0.82					
	1-02	26.7	2.98	1.12			7.31	2.74			2.73	1.02					
	1-03	27.9	3.24	1.16			6.10	2.18			3.46	1.24					
	1-04	23.7	2.79	1.18			5.74	2.42			3.47	1.47			vs coldMn		
	1-05	17.6	1.75	0.99	1.03	79.0	3.58	2.03	2.31	99.6	2.29	1.30	1.17	110.1	0.5649	0.3121	0.3437
					0.09	6.9			0.13	5.4			0.11	10.7			
Mn56x1-3D	2-01	23.9	3.59	1.50			6.97	2.92			3.56	1.49					
	2-02	21.5	1.44	0.67			3.90	1.81			2.42	1.12					
	2-03	12.4	1.12	0.90			2.66	2.14			1.68	1.35					
	2-04	15.0	1.60	1.07			3.03	2.03			1.59	1.06					
	2-05	23.7	2.36	1.00	1.03	78.9	3.23	1.36	2.05	88.7	2.11	0.89	1.18	111.6	0.5857	0.8237	0.8807
					0.14	10.5			0.25	11.0			0.11	10.0			
Mn56x3-3D	3-01	23.2	1.76	0.76			4.36	1.88			1.78	0.77					
	3-02	7.9	0.57	0.72			1.85	2.34			0.31	0.39					
	3-03	36.7	2.88	0.79			8.51	2.32			2.57	0.70					
	3-04	34.1	2.78	0.81			5.88	1.72			3.93	1.15					
	3-05	19.2	2.78	1.45	0.90	69.6	3.45	1.80	2.01	87.0	3.66	1.91	0.98	92.6	0.3388	0.8957	0.7803
					0.14	10.5			0.13	5.7			0.26	24.6			
Co60-3D	4-01	23.8	1.96	0.82			4.91	2.06			2.59	1.09					
	4-02	31.8	2.84	0.90			8.76	2.76			4.02	1.27					
	4-03	24.2	2.54	1.05			6.36	2.63			3.00	1.24					
	4-04	33.8	2.41	0.71			5.87	1.73			2.33	0.69			vs Control		
	4-05	13.7	1.22	0.89	0.87	67.2	2.72	2.00	2.24	96.6	1.40	1.03	1.06	100.1	0.0322	0.6001	0.4981
					0.06	4.2			0.20	8.5			0.10	9.7			
coldMn-3D	5-01	20.0	2.93	1.46			4.20	2.10			2.18	1.09					
	5-02	31.5	3.06	0.97			6.59	2.09			2.39	0.76					
	5-03	39.5	2.56	0.65			6.32	1.60			2.21	0.56					
	5-04	20.3	2.12	1.04			4.76	2.34			1.89	0.93					
	5-05	31.4	4.26	1.36	1.10	84.3	7.35	2.34	2.10	90.6	3.01	0.96	0.86	81.0			
					0.15	11.1			0.14	5.8			0.09	8.6			
C-3D	6-01	34.9	4.59	1.31			9.13	2.61			3.99	1.14					
	6-02	27.8	3.27	1.18			7.09	2.55			3.31	1.19					
	6-03	31.1	4.34	1.39			7.44	2.39			3.56	1.14					
	6-04	27.2	4.88	1.80			6.97	2.57			3.18	1.17					
	6-05	22.3	1.84	0.82	1.30	100.0	3.23	1.45	2.31	100.0	1.46	0.66	1.06	100.0			
					0.16	12.1			0.22	9.5			0.10	9.6			
**Day 14**					M/SE	%M/SE			M/SE	%M/SE			M/SE	%M/SE	DunnetTest		
Mn56x0.3-14D	1-06	27.4	1.20	0.44			3.67	1.34			1.47	0.54			AQP1	AQP5	Smad7
	1-07	17.5	1.23	0.70			2.55	1.45			1.11	0.63					
	1-08	25.8	2.12	0.82			3.59	1.39			3.17	1.23					
	1-09	16.8	1.13	0.67			2.27	1.35			1.40	0.83			vs coldMn		
	1-10	10.9	0.76	0.70	0.67	85.4	1.50	1.37	1.38	108.7	1.24	1.13	0.87	74.7	0.5166	0.4315	0.7159

					0.06	8.0			0.02	1.5			0.14	11.6			
Mn56x1-14D	2-06	24.0	1.91	0.79			5.13	2.13			1.60	0.67					
	2-07	13.2	1.04	0.79			1.86	1.41			1.69	1.28					
	2-08	12.0	1.24	1.04			2.18	1.82			2.12	1.77					
	2-09	20.1	1.51	0.75			2.77	1.38			2.23	1.11					
	2-10	17.3	1.25	0.72	0.82	105.0	2.21	1.27	1.60	126.0	1.99	1.15	1.20	102.6	0.0375	0.1875	0.1675
					0.06	7.2			0.16	12.8			0.18	15.2			
Mn56x3-14D	3-06	31.1	3.04	0.98			5.43	1.75			3.75	1.21					
	3-07	36.6	2.59	0.71			5.09	1.39			3.35	0.92					
	3-08	33.7	4.22	1.25			6.83	2.03			5.29	1.57					
	3-09	18.5	1.47	0.80			2.65	1.43			2.08	1.12					
	3-10	14.2	0.95	0.67	0.88	112.9	2.27	1.60	1.64	128.9	1.40	0.99	1.16	99.5	0.0878	0.0675	0.1102
					0.11	13.7			0.12	9.2			0.11	9.8			
Co60-14D	4-06	27.5	3.22	1.17			4.10	1.49			2.65	0.96					
	4-07	19.0	1.69	0.89			3.18	1.67			2.26	1.19					
	4-08	35.4	2.71	0.76			6.55	1.85			3.34	0.94					
	4-09	16.3	1.29	0.79			2.50	1.54			1.42	0.87			vs Control		
	4-10	14.3	0.90	0.63	0.85	109.1	2.25	1.58	1.63	127.8	1.05	0.74	0.94	80.7	0.2963	0.0728	0.0862
					0.09	11.6			0.06	4.9			0.07	6.3			
coldMn-14D	5-06	22.9	1.72	0.75			3.61	1.58			2.43	1.06					
	5-07	15.1	0.70	0.46			1.82	1.21			1.02	0.67					
	5-08	19.3	1.12	0.58			2.58	1.34			1.83	0.95					
	5-09	18.3	1.06	0.58			2.31	1.26			1.95	1.07					
	5-10	19.5	1.34	0.69	0.61	78.5	2.32	1.19	1.31	103.3	0.92	0.47	0.84	72.4			
					0.05	6.4			0.07	5.5			0.12	10.1			
C-14D	6-06	22.7	1.67	0.73			3.71	1.63			2.62	1.15					
	6-07	16.6	1.64	0.99			2.59	1.56			2.53	1.52					
	6-08	11.7	0.74	0.63			0.80	0.68			1.59	1.35					
	6-09	19.1	1.81	0.95			2.87	1.50			1.89	0.99					
	6-10	18.9	1.13	0.60	0.78	100.0	1.85	0.98	1.27	100.0	1.54	0.81	1.17	100.0			
					0.08	10.2			0.19	14.7			0.13	10.7			
**Day 70**					M/SE	%M/SE			M/SE	%M/SE			M/SE	%M/SE	DunnetTest		
Mn56x0.3-70D	1-11	18.3	1.13	0.62			2.96	1.62			1.59	0.87			AQP1	AQP5	Smad7
	1-12	28.4	2.37	0.83			4.74	1.67			3.46	1.22					
	1-13	16.4	1.07	0.65			2.38	1.45			1.76	1.07					
	1-14	19.5	1.73	0.89			4.45	2.28			2.35	1.20					
	1-15	29.7	3.29	1.11			6.31	2.13			3.41	1.15			vs coldMn		
	1-16	23.9	1.97	0.82	0.82	136.7	4.16	1.74	1.81	127.3	2.68	1.12	1.11	106.4	0.0286	0.2369	0.582
					0.07	12.0			0.13	9.2			0.05	5.0			
Mn56x1-70D	2-12	17.9	1.52	0.85			3.09	1.73			1.38	0.77					
	2-13	28.5	2.55	0.90			4.26	1.50			2.51	0.88					
	2-14	27.8	2.85	1.02			5.19	1.87			2.53	0.91					
	2-15	28.7	2.69	0.94			6.29	2.19			2.76	0.96					
	2-16	16.0	2.15	1.34	1.01	168.1	3.48	2.18	1.89	132.7	2.58	1.61	1.03	98.9			
					0.09	14.6			0.13	9.3			0.15	14.4	0.0085	0.1382	0.766

Mn56x3-70D	3-11	27.3	4.24	1.56			8.38	3.08			3.79	1.39					
	3-12	28.5	2.65	0.93			5.24	1.84			3.70	1.30					
	3-13	21.9	2.52	1.15			5.66	2.59			3.18	1.45					
	3-14	20.1	1.80	0.89			4.29	2.14			2.56	1.27					
	3-15	17.5	1.75	1.00			2.50	1.43			1.91	1.09					
	3-16	16.7	1.61	0.97	1.08	180.4	2.29	1.37	2.07	145.6	2.50	1.50	1.33	128.4	0.0049	0.159	0.0937
					0.10	16.8			0.27	19.2			0.06	5.8			
Co60-70D	4-11	33.6	1.90	0.56			7.04	2.09			3.92	1.17					
	4-12	39.2	2.66	0.68			6.84	1.74			5.33	1.36					
	4-13	20.1	1.65	0.82			4.57	2.27			3.24	1.61					
	4-14	32.0	1.64	0.51			5.00	1.56			3.69	1.15					
	4-15	20.1	0.96	0.48			2.57	1.28			1.44	0.72			vs Control		
	4-16	22.3	1.33	0.60	0.61	101.3	3.06	1.38	1.72	120.8	1.81	0.81	1.14	109.3	0.4676	0.1053	0.2713
					0.05	8.5			0.16	11.3			0.14	13.1			
coldMn-70D	5-11	32.7	1.84	0.56			4.75	1.46			3.17	0.97					
	5-12	47.9	2.27	0.47			6.04	1.26			3.21	0.67					
	5-13	27.5	1.72	0.63			4.54	1.65			2.88	1.05					
	5-14	19.2	1.04	0.54			3.18	1.65			1.80	0.93					
	5-15	30.3	1.65	0.54			3.76	1.24			3.58	1.18					
	5-16	29.7	2.17	0.73	0.58	96.5	6.29	2.12	1.56	109.7	4.70	1.59	1.06	102.4			
					0.04	6.1			0.13	9.4			0.12	12.0			
C-70D	6-11	28.6	1.15	0.40			2.73	0.95			2.91	1.02					
	6-12	42.3	2.50	0.59			6.31	1.49			4.67	1.10					
	6-13	26.3	1.62	0.62			3.02	1.15			2.23	0.85					
	6-14	23.8	1.29	0.54			4.16	1.75			2.36	0.99					
	6-15	25.7	1.54	0.60			3.34	1.30			2.37	0.92					
	6-16	31.2	2.68	0.86	0.60	100.0	5.96	1.91	1.42	100.0	4.23	1.35	1.04	100.0			
					0.06	10.1			0.15	10.4			0.07	6.9			
